# Significant value of ^18^F-FDG-PET/CT in diagnosing small cervical lymph node metastases in patients with nasopharyngeal carcinoma treated with intensity-modulated radiotherapy

**DOI:** 10.1186/s40880-017-0265-9

**Published:** 2017-12-19

**Authors:** Hao Peng, Lei Chen, Ling-Long Tang, Wen-Fei Li, Yan-Ping Mao, Rui Guo, Yuan Zhang, Li-Zhi Liu, Li Tian, Xu Zhang, Xiao-Ping Lin, Ying Guo, Ying Sun, Jun Ma

**Affiliations:** 1 Department of Radiation Oncology, State Key Laboratory of Oncology in South China, Collaborative Innovation Center for Cancer Medicine, Sun Yat-sen University Cancer Center, Guangzhou, 510060 Guangdong P. R. China; 2 Imaging Diagnosis and Interventional Center, State Key Laboratory of Oncology in South China, Collaborative Innovation Center for Cancer Medicine, Sun Yat-sen University Cancer Center, Guangzhou, 510060 Guangdong P. R. China; 3 Department of Nuclear Medicine, State Key Laboratory of Oncology in South China, Collaborative Innovation Center for Cancer Medicine, Sun Yat-sen University Cancer Center, Guangzhou, 510060 Guangdong P. R. China; 4 Department of Clinical Trials Center, State Key Laboratory of Oncology in Southern China, Collaborative Innovation Center for Cancer Medicine, Sun Yat-sen University Cancer Center, Guangzhou, 510060 Guangdong P. R. China

**Keywords:** Nasopharyngeal carcinoma, 18-fluoro-2-deoxy-glucose positron emission tomography with computed tomography (^18^F-PET/CT), Magnetic resonance image, Intensity-modulated radiotherapy, Small cervical lymph nodes

## Abstract

**Background:**

Little is known about the nature of metastasis to small cervical lymph nodes (SCLNs) in the patients with nasopharyngeal carcinoma (NPC) examined by using 18-fluoro-2-deoxy-glucose (^18^F-FDG) positron emission tomography/computed tomography (PET/CT). The present study aimed to evaluate the diagnostic values of PET/CT in identifying metastasis in SCLNs in NPC patients.

**Methods:**

Magnetic resonance images (MRI) and PET/CT scans for 470 patients with newly diagnosed, non-distant metastatic NPC were analyzed. Metastatic rates of SCLNs were defined by the positive number of SCLNs on PET/CT scans and total number of SCLNs on MRI scans. Receiver operating characteristic curve was applied to compare PET/CT-determined stage with MRI-determined stage.

**Results:**

In total, 2082 SCLNs were identified, with 808 (38.8%) ≥ 5 and < 6 mm in diameter (group A), 526 (25.3%) ≥ 6 and < 7 mm in diameter (group B), 374 (18.0%) ≥ 7 and < 8 mm in diameter (group C), 237 (11.4%) ≥ 8 and < 9 mm in diameter (group D), and 137 (6.5%) ≥ 9 and < 10 mm in diameter (group E). The overall metastatic rates examined by using PET/CT for groups A, B, C, D, and E were 3.5%, 8.0%, 31.3%, 60.0%, and 83.9%, respectively (*P* < 0.001). In level IV/Vb, the metastatic rate for nodes ≥ 8 mm was 84.6%. PET/CT examination resulted in modification of N category and overall stage for 135 (28.7%) and 46 (9.8%) patients, respectively. The areas under curve of MRI-determined and PET/CT-determined overall stage were 0.659 and 0.704 for predicting overall survival, 0.661 and 0.711 for predicting distant metastasis-free survival, and 0.636 and 0.663 for predicting disease-free survival.

**Conclusions:**

PET/CT was more effective than MRI in identifying metastatic SCLNs, and the radiologic diagnostic criteria for metastatic lymph nodes in level IV/Vb should be re-defined.

## Background

The Epstein-Barr virus (EBV)-associated malignancy nasopharyngeal carcinoma (NPC) has a geographically unbalanced distribution [1, 2] and is endemic in regions such as South China, where the incidence is 20–50 per 100,000 males [3]. Radiotherapy is the only definitive treatment option for NPC as a result of its high radiosensitivity and anatomic constraints that make surgery complex. NPC is also sensitive to chemotherapy; combined strategies such as concurrent chemoradiotherapy (CCRT) with or without adjuvant chemotherapy have been established as the standard care for advanced disease [4–6], whereas radiotherapy alone is adequate for early-stage disease.

Advances in imaging, diagnosis, and treatment over the last two decades have made NPC more curable. Undoubtedly, accurate staging workup is an essential component of these improvements. Magnetic resonance imaging (MRI) has replaced computed tomography (CT) as the most important staging tool, due to its excellent soft tissue contrast resolution and precise detection of early and deep primary tumor involvement [7]. Notably, more than 80% of patients present with lymph node metastasis at initial diagnosis [8, 9]. Besides, small cervical lymph nodes (SCLNs) that do not reach the radiologic criteria of 10 mm for metastatic lymph nodes [10] are also commonly observed. Radiologists and clinicians may be less likely to assess the nature of SCLNs based on MRI findings since nodal size and the presence of necrosis are the only references for nodal involvement on MRI [11]; however, overlooking SCLNs could impede precise staging and treatment.

In an effort to develop individualized and precise medicine approaches, 18-fluoro-2-deoxy-glucose (^18^F-FDG) positron emission tomography/computed tomography (PET/CT) is playing an increasingly important role in diagnosis of NPC. Numerous studies have documented that ^18^F-FDG PET/CT is superior to MRI for N categorization in both NPC and other head and neck cancers [12–16]. However, no studies have yet assessed the value of ^18^F-FDG PET/CT for diagnosing SCLNs in NPC. Given the urgent needs for precise staging and individualized treatment, we conducted this study to further assess the role of ^18^F-FDG PET/CT in the diagnosis of SCLNs in NPC.

## Methods

### Patients

We retrospectively analyzed the data on patients with newly diagnosed, non-metastatic NPC treated at Sun Yat-sen university center between November 2009 and May 2012. Patients meeting the following criteria were included in this study: (1) stage I–IVA disease; (2) age ≥ 18 years; (3) no previous malignancy; (4) receiving ^18^F-FDG PET/CT and MRI as pre-treatment staging workup at our center; and (5) treated with intensity-modulated radiotherapy (IMRT). Informed consent was obtained from all patients before treatment, and this study was approved by the institutional research ethics committee of Sun Yat-sen University Cancer Center.

### Staging workup

A complete medical history and clinical examinations of the head and neck region were performed for all patients at initial diagnosis. All participants received MRI and ^18^F-FDG PET/CT scans as the main staging workup. All patients were re-staged twice according to the 8th edition of the International Union against Cancer/American Joint Committee on Cancer (UICC/AJCC) staging system [17]: (1) for MRI-diagnosed stage, T and N categories were determined only based on MRI findings; (2) for PET/CT-diagnosed stage, T category was determined based on MRI findings, and N category was determined based on PET/CT findings.

### Measurement of small cervical lymph nodes

**Fig. 1:**
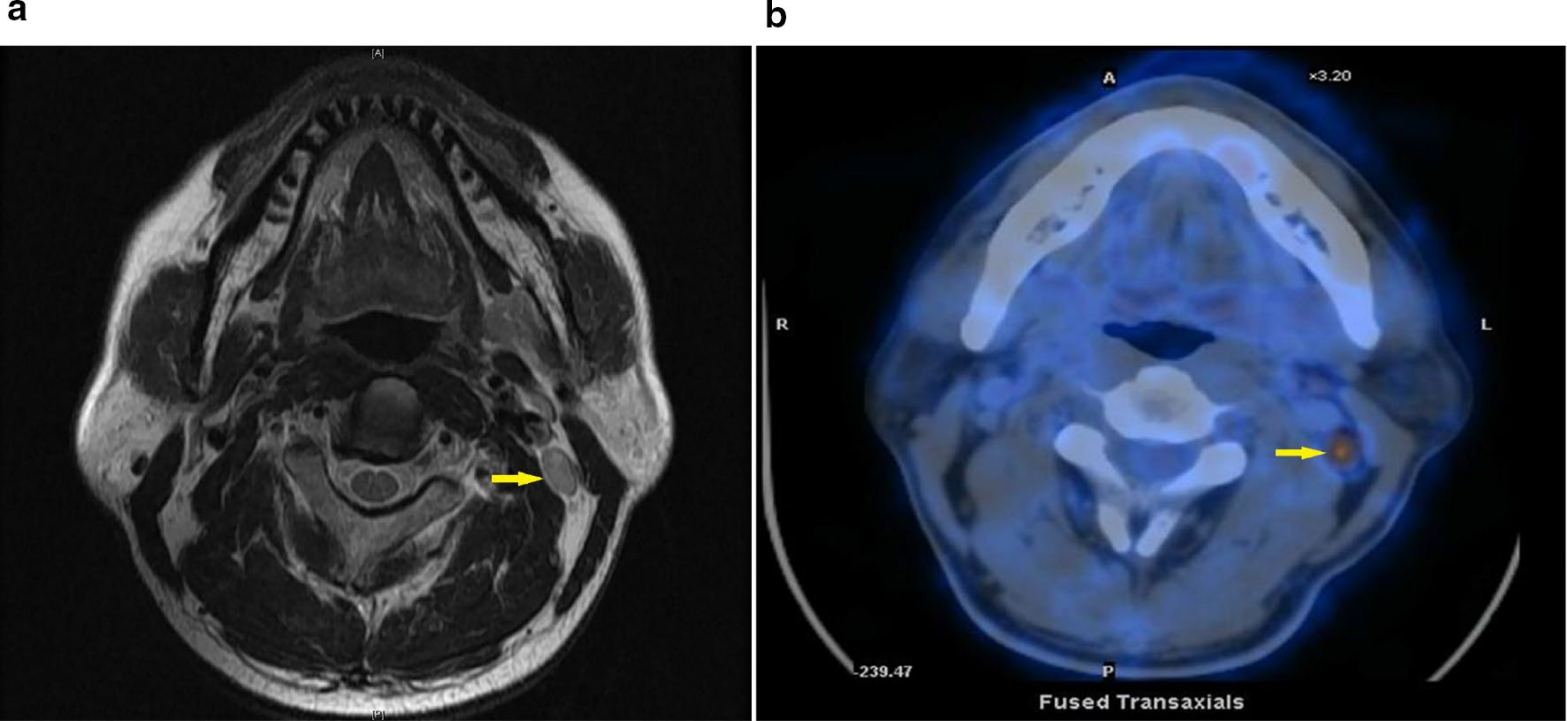
A 35-year-old man with a SCLN of 7.5 mm (arrow) in left level IIb was diagnosed with T3N0 nasopharyngeal cancer by MRI (**a**), but diagnosed with T3N1 disease by PET/CT (**b**). *SCLN* small cervical lymph node, *MRI* magnetic resonance imaging, *PET/CT* positron emission tomography/computed tomography

In this study, only cervical lymph nodes with a short-axis diameter ≥ 5 and < 10 mm (Fig. 1) were included and were divided into five groups according to the diameter: (A) ≥ 5 and < 6 mm; (B) ≥ 6 and < 7 mm; (C) ≥ 7 and < 8 mm; (D) ≥ 8 and < 9 mm; and (E) ≥ 9 and < 10 mm. Retropharyngeal lymph nodes were not included in this analysis, but were analyzed for N category classification and overall stage modification. Lymph nodes with a short-axis diameter in the ranges listed above but displayed necrosis were not classified as SCLNs because necrosis is one of the criteria for metastatic lymph node. Two radiologists (L.Z.L. and L.T.) with more than 10-year experience employed at our hospital separately evaluated all images and measured the short-axis diameters of the lymph nodes without reference to PET/CT findings. Disagreements were resolved by consensus.

### PET/CT imaging

In our hospital, PET/CT would be recommended if patients were diagnosed with N2-3 NPC by MRI or patients asked for this examination. Patients fasted for at least 6 h before the PET/CT scanning; patients with fasting plasma glucose > 2 g/L were excluded from this study. The PET/CT scanner (Discovery ST 16; GE Healthcare, Little Chalfont, United Kingdom) was employed to obtain PET/CT images according to the guidelines for tumor imaging with PET/CT [18]. PET/CT scans were initiated 45–60 min after injection of 5.55 MBq/kg ^18^F-FDG. Two doctors of nuclear medicine (X.Z. and X.P.L.) with more than 10-year experience at our center separately evaluated all small lymph nodes based on standardized uptake value (SUV) without reference to the MRI findings, and any disagreements were resolved by consensus. Usually, small lymph nodes with abnormal uptake of ^18^F-FDG were regard as metastatic lymph nodes. Notably, the diagnostic criteria of SUV for metastatic lymph nodes is about 2.5 [19].

### Treatment

All participants received IMRT at our hospital. A total prescribed dose of 66–72 Gy at 2.12–2.43 Gy/fraction was delivered to the planning target volume (PTV) of the gross tumor volume of the nasophrynx (GTVnx), 64–70 Gy to the PTV of the GTV of the metastatic lymph nodes (GTVnd), 60–68 Gy to the PTV of the GTV of the SCLNs (GTVs), 60–63 Gy to the PTV of the high-risk clinical target volume (CTV1), and 54–56 Gy to the PTV of the low-risk clinical target volume (CTV2). The simultaneous integrated boost technique was adopted to treat all targets.

We recommended radiotherapy alone for stage I disease, radiotherapy with or without concurrent chemotherapy for stage II disease, and CCRT with or without neoadjuvant chemotherapy (NCT) for stage III-IVB disease. NCT consisted of cisplatin (80 mg/m^2^ d1) with 5-fluorouracil (1000 mg/m^2^ on days 1–5) or cisplatin (75 mg/m^2^ on day 1) with docetaxel (75 mg/m^2^ on day 1) every 3 weeks for two to four cycles. Concurrent chemotherapy was to administer cisplatin weekly (30–40 mg/m^2^) or tri-weekly (80–100 mg/m^2^).

### Follow-up and statistical analysis

Follow-up was measured from the first day of treatment to last visit or death. Patients were assessed every 3 months during the first 2 years and every 6 months thereafter. Locoregional or distant failures were confirmed by pathology or imaging methods. The primary endpoint was the metastatic rate of SCLN diagnosed by using PET/CT. Other endpoints included overall survival (OS; time from treatment initiation to death from any cause or the last follow-up), distant metastasis-free survival (DMFS; time from treatment initiation to distant failure or the last follow-up), and disease-free survival (DFS; time from treatment initiation to treatment failure or death or the last follow-up). Kaplan–Meier method was adopted to calculate survival rates, and difference was compared by log-rank test. Patients lost to follow-up would be treated as censored data and were culled by Kaplan–Meier method.

The Chi square test was used to compare the metastatic rates of SCLNs between different groups (group A–E) and cervical levels (level I–V). Receiver operating characteristic (ROC) curve analysis based on 3-year OS, DMFS, and DFS was used to compare MRI-determined tumor stage with that diagnosed by PET/CT. All tests were two-sided; *P* < 0.05 was considered significant. Stata Statistical Package 12 (StataCorp LP, College Station, TX, USA) was used for all analyses.

## Results

### Baseline information

Finally, we reviewed the data of 2191 patients, and 470 (21.5%) of them were eligible for this study. Of the 470 patients included in this study, 358 (76.2%) were male and 112 (23.8%) were female, carrying a ratio of 3.2:1. Median age was 46 (range 16–77) years. Baseline characteristics are summarized in Table 1. In total, 126 (26.9%) patients had early-stage (stage I–II) diseases, and 68 (14.5%) patients did not receive any chemotherapy. By November 2016, 29 (6.2%) patients were lost to follow-up; median follow-up was 62.9 (range 3.7–91.5) months. Overall, 98 (20.9%) patients experienced treatment failure.

**Table 1:** Baseline characteristics of the 470 patients with nasopharyngeal carcinoma

Characteristic	Number of patients	Percentage (%)
Gender		
Male	358	76.2
Female	112	23.8
Smoking		
Yes	170	36.2
No	300	63.8
Alcohol consumption		
Yes	60	12.8
No	410	87.2
Family history of cancer		
Yes	152	32.3
No	318	67.7
MRI-based stage		
T category^a^		
T1	77	16.4
T2	81	17.2
T3	239	50.9
T4	73	15.5
N category^a^		
N0	81	17.2
N1	279	59.4
N2	71	15.1
N3	39	8.3
Overall stage^a^		
I	28	6.0
II	98	20.9
III	239	50.9
IVa–b	105	22.2
Chemotherapy		
No	68	14.5
Neoadjuvant alone	50	10.6
Concurrent alone	146	31.1
Induction plus concurrent	206	43.8
Treatment failure		
Death	70	14.9
Distant metastasis	59	12.6
Locoregional recurrence	42	8.9

*MRI* magnetic resonance imaging

^a^According to the 8th UICC/AJCC staging system

### SCLN-metastatic rate according to PET/CT findings

Overall, 2082 SCLNs were identified, of which 808 (38.8%) were ≥ 5 and < 6 mm (group A), 526 (25.3%) were ≥ 6 and < 7 mm (B), 374 (18.0%) were ≥ 7 and < 8 mm (C), 237 (11.4%) were ≥ 8 and < 9 mm (D), and 137 (6.6%) were ≥ 9 and < 10 mm (E; Table 2). Most (1266/2082, 60.8%) of the SCLNs were observed in level II. Based on ^18^F-FDG PET/CT findings, the overall metastatic rates of SCLNs for groups A, B, C, D, and E were 3.5%, 8.0%, 31.3%, 60.0%, and 83.9%, respectively. The metastatic rate significantly increased as nodal size increased from 5 to 10 mm at all levels (*P* < 0.001). The metastatic rates were usually very low for lymph nodes < 8 mm in level I and lymph nodes < 7 mm in levels II/III/IV/V. For group A and B nodes, the metastatic rates were similar between levels II, III/Va and IV/Vb (*P* = 0.768) but significantly higher than those at level I (*P* = 0.018). For group C and D nodes, the metastatic rates were significantly increased from level I to IV/Vb (*P* < 0.001 for group C and *P* = 0.001 for group D). However, the metastatic rates for group E nodes were not significantly different between all levels (*P* = 0.487).

**Table 2:** Metastatic rates of small cervical lymph nodes based on MRI and PET/CT findings

Level	Group A (≥ 5 and < 6 mm)		Group B (≥ 6 and < 7 mm)		Group C (≥ 7 and < 8 mm)		Group D (≥ 8 and < 9 mm)		Group E (≥ 9 and < 10 mm)		*P* ^e^
	Total^a^	Metastatic lymph nodes (%)^b^	Total^a^	Metastatic lymph nodes (%)^b^	Total^a^	Metastatic lymph nodes (%)^b^	Total^a^	Metastatic lymph nodes (%)^b^	Total^a^	Metastatic lymph nodes (%)^b^	
I^c^	197	0 (0)	70	0 (0)	34	2 (5.9)	10	2 (20.0)	3	3 (100.0)	< 0.001
II	396	17 (4.3)	329	30 (9.1)	263	80 (30.4)	167	94 (56.3)	111	91 (82.0)	< 0.001
III/Va	170	8 (4.7)	91	8 (8.8)	58	23 (39.7)	34	24 (70.6)	12	11 (91.7)	< 0.001
IV/Vb	45	3 (6.7)	36	4 (11.1)	19	12 (63.2)	26	22 (84.6)	11	10 (90.9)	< 0.001
*P*_1_^d^	0.018		0.005		< 0.001		0.001		0.487		
*P*_2_^d^	0.768		0.915		0.008		0.011		0.503		

*MRI* magnetic resonance imaging, *PET/CT* positron emission tomography/computed tomography

^a^Total number of small cervical lymph nodes based on MRI findings

^b^The number of metastatic small cervical lymph nodes based on PET/CT findings

^c^There were 184 (93.4%) in the level Ib and 13 (6.6%) in the level Ia for group A. No small cervical lymph nodes were observed in level Ia for the other groups

*P*_1_^d^: Comparison between levels I, II, III/Va, and IV/Vb; *P*_2_^d^: Comparison between levels II, III/Va, and IV/Vb

^e^Comparison between groups A, B, C, D and E. P values are calculated by Chi square test or Fisher’s exact test

### Subgroup analysis stratified by N category

As the tumor burden significantly increases from N0 to N3 diseases, the metastatic rates for each SCLN group may vary between N categories. We therefore assessed the impact of MRI-diagnosed N category on the metastatic rates of SCLN (Table 3). Consistent with the results above, the metastatic rates significantly increased with the nodal size in almost all N-category subsets, except for those at levels I and IV/Vb in N0 disease (*P* = 1.000). With regards to groups A and B, the metastatic rates were comparable between levels II, III/Va and IV/Vb (*P* > 0.05 for all groups), but higher than that of level I nodes (*P* = 0.008 for group A and *P* < 0.001 for group B). Generally, the metastatic rates increased from level I to IV/Vb in groups C and D; however, these differences were not significant (*P* > 0.05 for all rates). Intriguingly, the metastatic rates for group E nodes were similar between the five cervical levels.

**Table 3:** Metastatic rates of small cervical lymph nodes based on MRI and PET/CT findings stratified by N category

MRI-based N category	Group A		Group B		Group C		Group D		Group E		*P* ^*c*^
	Total^a^	Metastatic lymph nodes (%)^b^	Total^a^	Metastatic lymph nodes (%)^b^	Total^a^	Metastatic lymph nodes (%)^b^	Total^a^	Metastatic lymph nodes (%)^b^	Total^a^	Metastatic lymph nodes (%)^b^	
N0 category											
Level I	37	0 (0)	9	0 (0)	4	0 (0)	1	0 (0)	0	0 (0)	1.000
Level II	75	0 (0)	47	0 (0)	37	4 (10.8)	17	7 (41.2)	7	6 (85.7)	< 0.001
Level III/Va	16	0 (0)	8	1 (12.5)	1	0 (0)	2	1 (50.0)	1	1 (100.0)	0.036
Level IV/Vb	6	0 (0)	3	0 (0)	1	0 (0)	0	0 (0)	0	0 (0)	1.000
*P*_1_^c^	1.000		0.164		1.000		1.000		1.000		
*P*_2_^c^	1.000		0.190		1.000		0.812		1.000		
N1 category											
Level I	115	0 (0)	41	0 (0)	21	0 (0)	7	1 (14.3)	2	2 (100.0)	< 0.001
Level II	239	8 (3.3)	189	34 (18.0)	168	54 (32.1)	95	54 (56.8)	68	56 (82.4)	< 0.001
Level III/Va	85	7 (8.2)	52	3 (5.8)	25	8 (32.0)	13	9 (69.2)	5	5 (100.0)	< 0.001
Level IV/Vb	19	1 (5.3)	17	2 (11.8)	8	4 (50.0)	9	7 (77.8)	4	4 (100.0)	< 0.001
*P*_1_^c^	0.008		< 0.001		0.001		0.045		0.746		
*P*_2_^c^	0.147		0.056		0.592		0.341		0.647		
N2 category											
Level I	22	0 (0)	16	0 (0)	6	1 (16.7)	1	0 (0)	1	1 (100.0)	0.013
Level II	66	6 (9.1)	67	8 (11.9)	44	17 (38.6)	35	21 (60.0)	27	21 (77.8)	< 0.001
Level III/Va	54	1 (1.9)	20	4 (20.0)	18	8 (44.4)	10	7 (70.0)	2	2 (100.0)	< 0.001
Level IV/Vb	8	1 (12.5)	4	0 (0)	2	0 (0)	3	3 (100.0)	1	1 (100.0)	0.007
*P*_1_^c^	0.151		0.305		0.441		0.130		1.000		
*P*_2_^c^	0.135		0.593		0.623		0.074		1.000		
N3 category											
Level I	23	0 (0)	4	0 (0)	3	1 (33.3)	1	1 (100.0)	0	0 (0)	0.013
Level II	16	3 (18.8)	26	7 (26.9)	14	5 (35.7)	20	12 (60.0)	9	8 (88.9)	< 0.001
Level III/Va	15	0 (0)	11	0 (0)	14	7 (50.0)	9	7 (77.8)	4	3 (75.0)	< 0.001
Level IV/Vb	12	1 (8.3)	12	2 (16.7)	8	8 (100.0)	14	12 (85.7)	6	5 (83.3)	< 0.001
*P*_1_^c^	0.038		0.232		0.006		0.380		1.000		
*P*_2_^c^	0.117		0.06		0.003		0.225		1.000		

*MRI* magnetic resonance imaging, *PET/CT* positron emission tomography/computed tomography

^a^Total number of small cervical lymph nodes based on MRI findings

^b^Metastatic number of small cervical lymph nodes based on PET/CT findings

^c^ *P* values were calculated using the Chi square test or Fisher’s exact test if indicated; *P*_1_^c^: Comparison between levels I, II, III/Va and IV/Vb; *P*_2_^c^: Comparison between levels II, III/Va and IV/Vb

### Stage modification

Based on PET/CT findings, 135 (28.7%) and 46 (9.8%) patients required N category and overall stage modifications, respectively (Table 4). Specifically, 86 (18.3%) patients required an increase in N category: 17 (3.6%) from N0 to N1, 2 (0.4%) from N0 to N2, 47 (10.0%) from N1 to N2, 15 (3.2%) from N1 to N3, and 5 (1.0%) from N2 to N3. Forty-nine (10.4%) patients required a reduction in N category: 43 (9.1%) from N1 to N0, 1 (0.2%) from N2 to N0, and 5 (1.0%) from N2 to N1. Consequently, PET/CT examination led to up-staging for 34 (7.2%) patients including 5 (1.0%) from stage I to II, 12 (2.6%) from II to III, 4 (0.9%) from II to IV, and 13 (2.8%) from III to IV, and down-staging for 12 (2.6%) patients including 2 (0.4%) from stage III to II, and 10 (2.1%) from II to I.

**Table 4:** Comparison of MRI- and PET/CT-determined tumor staging

Tumor stage	MRI-determined staging				PET/CT-determined staging			
	N0	N1	N2	N3	N0	N1	N2	N3
T1	28 (6.0)	39 (8.3)	8 (1.7)	2 (0.4)	33 (7.0)	30 (6.4)	10 (2.1)	4 (0.9)
T2	18 (3.8)	41 (8.7)	13 (2.8)	9 (1.9)	21 (4.5)	28 (6.0)	18 (3.8)	14 (3.0)
T3	28 (6.0)	153 (32.6)	37 (7.9)	21 (4.5)	45 (9.6)	104 (22.1)	59 (12.6)	31 (6.6)
T4	7 (1.5)	46 (9.8)	13 (2.8)	7 (1.5)	7 (1.5)	34 (7.2)	22 (4.7)	10 (2.1)
Stage I	28 (6.0)				33 (7.0)			
Stage II	98 (20.9)				79 (16.8)			
Stage III	239 (50.8)				236 (50.2)			
Stage IV	105 (22.3)				122 (26.0)			

All values are presented as number of patients followed by percentage in parentheses

*MRI* magnetic resonance imaging, *PET/CT* positron emission tomography/computed tomography

### Comparison of MRI- and PET/CT-based tumor staging

**Fig. 2:**
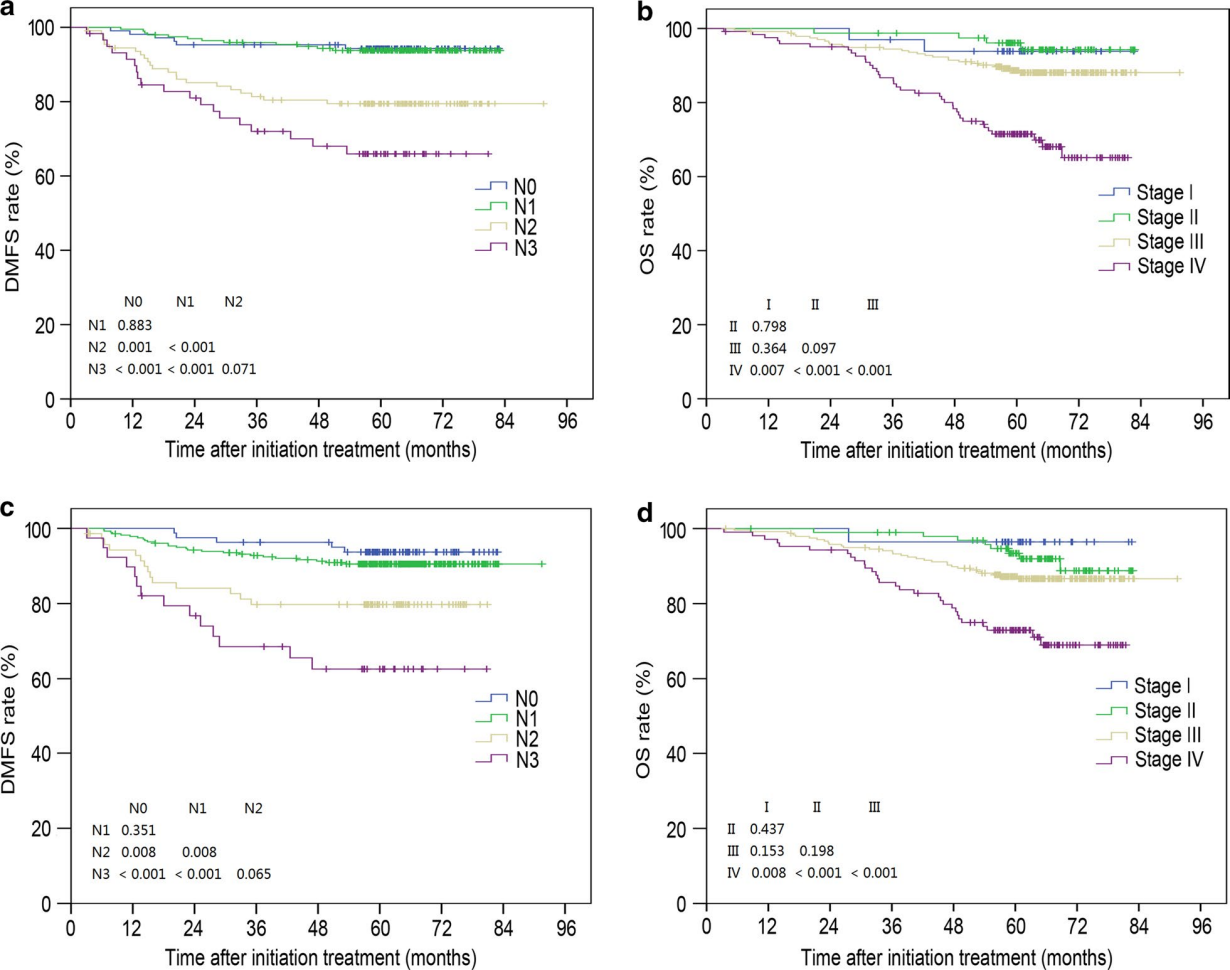
Kaplan-Meier DMFS and OS curves stratified by MRI-diagnosed N category (**a**) and overall stage (**b**), and PET/CT-diagnosed N category (**c**) and overall stage (**d**). Numbers in the figure are *P* values. *DMFS* distant metastasis-free survival, *OS* overall survival, *MRI* magnetic resonance imaging, *PET/CT* positron emission tomography/computed tomography

**Fig. 3:**
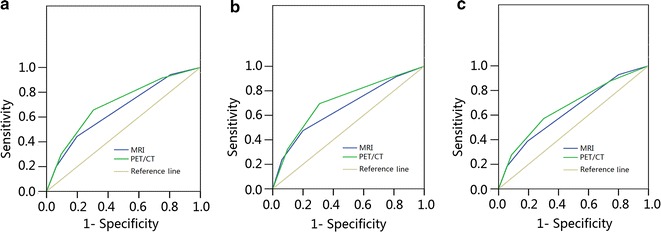
ROC curve analysis comparing MRI-determined and PET/CT-determined overall stage for **a** OS, **b** DMFS, and **c** DFS. *ROC* Receiver operating characteristic, *MRI* magnetic resonance imaging, *PET/CT* positron emission tomography/computed tomography, *OS* overall survival, *DMFS* distant metastasis-free survival, *DFS* disease-free survival

Univariate analysis of survival outcomes of patients with different N category and overall stage based on MRI- and PET/CT findings is presented in Fig. 2. Compared with MRI-diagnosed tumor stage (Fig. 2a, b), PET/CT-diagnosed tumor stage provided better stratification of OS and DMFS (Fig. 2c, d). ROC curve analysis was performed to directly compare the superiority of overall stage based on MRI and PET/CT findings (Fig. 3). With respect to 3-year OS (Fig. 3a), the area under curve (AUC) was 0.659 for MRI-diagnosed stage (AUC_MRI_) and 0.704 for PET/CT-diagnosed stage (AUC_PET/CT_). The AUC_MRI_ and AUC_PET/CT_ values were 0.661 and 0.711 for 3-year DMFS (Fig. 3b) and 0.636 and 0.663 for 3-year DFS (Fig. 3c), respectively. Therefore, PET/CT-diagnosed tumor stage was superior to MRI-diagnosed stage in terms of prognostic stratification.

## Discussion

In our present study, we focused on SCLNs in NPC patients and revealed that the metastatic rates of SCLNs assessed based on PET/CT findings significantly increased with nodal size, regardless of cervical level. Further subgroup analysis stratified by N category demonstrated similar trends. In addition, ROC curve analysis revealed that PET/CT-diagnosed tumor stage provides superior prognostic stratification compared with MRI-diagnosed tumor stage.

^18^F-FDG PET/CT imaging provides an important contribution to the diagnosis of cancer and prediction of prognosis due to that it adds tissue functional information. Numerous studies have demonstrated that the maximum SUV in PET/CT imaging could serve as a valuable prognostic factor in NPC [19–24]. Moreover, PET/CT has been proved to be more accurate than conventional CT/MR imaging for detection of nodal metastasis in both NPC [14, 15] and other head and neck cancers [12, 13, 25, 26]. In the present study, PET/CT was also superior to MRI for diagnosing metastasis in SCLNs with diameters less than 10 mm, thereby resulting in better tumor stage stratification. Similarly, PET/CT improved staging in 15%–20% of cases of other head and neck cancer [25, 27].

As a distinct head and neck cancer, NPC has a very high incidence of lymph node metastasis, ranging from 85% to 86.4% [8, 9, 28]. Treatment guidelines have been proposed for metastatic lymph nodes, with a recommended radical radiation dose of 66–70 Gy. However, little attention has been paid to SCLNs, and no treatment consensus has yet been achieved. This situation may be attributed to the uncertainty of the nature of these lymph nodes based on MRI findings, since NPC is typically treated without pathologic assessment of the cervical lymph nodes. Recently, two retrospective studies found that SCLNs did not affect prognosis of patients [28, 29]; however, the precise details of the radiation doses delivered to the SCLNs were not provided in either study. In fact, treatment of SCLNs is mainly based on individual clinicians’ experience. Cervical lymph node status is the main factor that affects distant control and OS of NPC patients [30]. Therefore, correct identification of the nature of SCLNs is essential for individualized treatment planning. In the present study, we subdivided SCLNs into five groups based on maximal diameter and presented the corresponding metastatic rates for each group as determined according to PET/CT findings. For nodes smaller than 7 mm, the metastatic rates were very low at all levels. Therefore, a radical radiation dose may be inappropriate for these lymph nodes. With regards to nodes ≥ 7 and < 9 mm, the metastatic rates significantly increased from level I–IV/Vb, with the highest metastatic rate of 84.6% in level IV/Vb. Thus, a radical dose may be necessary, especially for nodes in the lower cervical region. For nodes ≥ 9 mm, the overall metastatic rate reached 84%, and was over 90% in levels III/Va and IV/Vb. Hence, a radical radiation dose should be delivered. Generally, the results of the subgroup analysis were similar to those of the primary analysis. Therefore, it is unlikely that N category affects the metastatic rates of SCLNs for different node size groups and neck levels.

Notably, only SCLNs with a diameter ≥ 5 mm were included in this analysis, mainly for three reasons. First, the retropharyngeal nodes are the first-echelon chain of lymph node metastasis in NPC [31, 32] and have a radiologic diagnostic criteria of 5 mm for metastasis [33]. Therefore, the cervical regions may be non-metastatic for SCLNs less than 5 mm since they are not the first station of node metastasis. Second, the size threshold for accurate detection of metastatic cervical lymph nodes using ^18^F-FDG PET/CT is 5 mm [34, 35]. Moreover, a large measurement error in the diameter of such small lymph nodes may exist. Given these reasons, we therefore selected the threshold of 5 mm. It is important to note that we did not include small retropharyngeal lymph nodes (< 5 mm), as retropharyngeal nodes are adjacent to the primary tumor and are treated as part of the GTVnx, regardless of nodal size.

Based on the results of the present study, we suggest the current radiologic diagnosis criteria of 10 mm for metastatic cervical level IV/Vb lymph nodes may be too strict; 8 mm may be more appropriate since the metastatic rate for nodes > 8 mm was over 80%. Moreover, nodes ≥ 9 mm should be treated as metastases in clinical practice. However, the limitations of this study should be acknowledged. First, the data was collected retrospectively from patients treated at a single center. More importantly, pathologic assessment of SCLNs was not possible, as NPC—unlike other head and neck cancers—is typically treated without pathologic analysis of metastatic lymph nodes. Nevertheless, false negative/false positive results may occur due to micrometastases or necrosis, whereas false positive findings could be attributed to reactive changes and inflammation [10]. Despite these potential pitfalls, ^18^F-FDG PET/CT is still superior to MRI for identification of lymph node metastasis in NPC.

## Conclusions

^18^F-FDG PET/CT was more effective than MRI in diagnosing SCLNs with diameters ≥ 5 and < 10 mm. Metastatic rates significantly increased with the nodal size. Moreover, the diagnostic criteria for metastatic lymph nodes in level IV/Vb should be re-defined. These findings provide strong evidence for the clinical management of SCLNs. However, further studies including pathologic assessment are warranted to confirm our results.
